# MxaY regulates the lanthanide-mediated methanol dehydrogenase switch in *Methylomicrobium buryatense*

**DOI:** 10.7717/peerj.2435

**Published:** 2016-09-07

**Authors:** Frances Chu, David A.C. Beck, Mary E. Lidstrom

**Affiliations:** 1 Department of Chemical Engineering, University of Washington, Seattle, WA, United States; 2 eScience Institute, University of Washington, Seattle, WA, United States; 3 Department of Microbiology, University of Washington, Seattle, WA, United States

**Keywords:** Methanol dehydrogenase, XoxF, Lanthanides, MxaY, MxaFI

## Abstract

Many methylotrophs, microorganisms that consume carbon compounds lacking carbon–carbon bonds, use two different systems to oxidize methanol for energy production and biomass accumulation. The MxaFI methanol dehydrogenase (MDH) contains calcium in its active site, while the XoxF enzyme contains a lanthanide in its active site. The genes encoding the MDH enzymes are differentially regulated by the presence of lanthanides. In this study, we found that the histidine kinase MxaY controls the lanthanide-mediated switch in *Methylomicrobium buryatense* 5GB1C. MxaY controls the transcription of genes encoding MxaFI and XoxF at least partially by controlling the transcript levels of the orphan response regulator MxaB. We identify a constitutively active version of MxaY, and identify the mutated residue that may be involved in lanthanide sensing. Lastly, we find evidence to suggest that tight control of active MDH production is required for wild-type growth rates.

## Introduction

Interest in the use of methanotrophs, or methane-oxidizing bacteria, as biological catalysts for the production of chemicals or biofuels has increased as atmospheric levels of methane rise. Methanotrophs oxidize and utilize methane as their carbon and energy source, and the industrial utilization of methanotrophs is an attractive way to mitigate atmospheric methane (Fei et al., 2014; Haynes & Gonzalez, 2014; Kalyuzhnaya, Puri & Lidstrom, 2015). New genetic tools and the discovery of rapidly-growing methanotrophic strains has enabled rapid iteration of metabolic engineering strategies (Puri et al., 2015; Yan et al., 2016). The deployment of methanotrophs for industrial use is dependent on successful metabolic engineering strategies, which requires an in-depth knowledge of methanotrophic metabolism.

Methanotrophs are a subset of microorganisms that are capable of methylotrophy, the metabolic mode in which reduced carbon substrates containing no carbon–carbon bonds are converted into energy and biomass (Anthony, 1982; Lidstrom, 2006; Chistoserdova, 2011). In methanotrophs, reducing power is generated when methane is first oxidized to methanol and then to formaldehyde. Formaldehyde can be reduced further to formate and carbon dioxide to generate more reducing power, or it can be assimilated into biomass (Chistoserdova, 2011). Type I methanotrophs, which are gammaprotebacteria, assimilate formaldehyde into biomass using the highly efficient ribulose monophosphate (RuMP) pathway (Kemp & Quayle, 1966; Kemp & Quayle, 1967; Kalyuzhnaya et al., 2013). Type I methanotrophs are particularly suited for use as industrial microorganisms as this assimilatory pathway allows for the most efficient conversion of methane into value-added chemicals (Kalyuzhnaya, Puri & Lidstrom , 2015).

In the majority of methanotrophs identified, the first two reactions required to convert methane into biomass and energy are carried out by virtually functionally redundant enzymes, whose gene transcription is regulated by the metals contained in the enzyme active sites (Semrau, DiSpirito & Yoon, 2010; Chistoserdova, 2011; Keltjens et al., 2014; Farhan Ul Haque et al., 2015b; Chu & Lidstrom, 2016). Functional redundancy between these enzyme complexes may be crucial in fluctuating environments with uncertain metal availability. The tight transcriptional regulation of these enzyme complexes by active site metals suggests that the unnecessary production of inactive enzymes is metabolically costly to cells.

Methane oxidation in most methanotrophs can be carried out by two separate, differentially-regulated methane oxidation systems: the copper-dependent particulate methane oxidation (pMMO) system and the iron-dependent soluble methane oxidation (sMMO) system (Semrau, DiSpirito & Yoon, 2010; Chistoserdova, 2011). The presence of copper regulates the transcription of these two systems, with genes encoding pMMO being induced only in the presence of copper (Nielsen, Gerdes & Murrell, 1997; Choi et al., 2003). Under these conditions, the genes encoding the sMMO system are downregulated.

Similarly, methanol oxidation can be carried out by two differentially-regulated methanol dehydrogenase (MDH) enzymes. Both MDH enzymes are periplasmic and contain the pyrroloquinoline quinone (PQQ) cofactor. The canonical, well-studied MDH is a heterotetrameric enzyme composed of two large subunits, MxaF, and two small subunits, MxaI, with a calcium ion in the MxaF active site. The more recently-discovered XoxF MDH is reported to be composed of a homodimer of XoxF subunits (Harms et al., 1996; Chistoserdova & Lidstrom, 1997; Schmidt et al., 2010; Chistoserdova, 2011; Pol et al., 2014). Purified XoxF enzymes have been found to contain lanthanides, rather than calcium, in the active site (Pol et al., 2014).

Similar to copper regulation of genes encoding methane monooxygenases, lanthanides differentially regulate the transcription of genes encoding the MDH enzymes (Farhan Ul Haque et al., 2015b; Skovran & Martinez-Gomez, 2015; Chu & Lidstrom, 2016; Gu et al., 2016; Vu et al., 2016). The gene encoding XoxF is transcribed in the presence of lanthanides and the operon encoding the MxaFI genes is transcribed in the absence of lanthanides. Moreover, XoxF has been purified as the dominant MDH when methylotrophs are cultivated in the presence of lanthanides (Fitriyanto et al., 2011; Hibi et al., 2011; Nakagawa et al., 2012). As concentration of lanthanides in the environment is high enough to favor *xoxF* transcription, XoxF is likely the dominant MDH operant under environmental conditions in some methylotrophic species (Delmotte et al., 2009; Grob et al., 2015; Ramachandran & Walsh, 2015; Skovran & Martinez-Gomez, 2015; Chu & Lidstrom, 2016; Vu et al., 2016).

Numerous regulators have been found to control the transcription of genes encoding the MDH enzymes. In the non-methanotrophic methylotroph *Methylobacterium extorquens* AM1, the *mxa* operon is controlled by at least two two-component systems, MxcQE and MxbDM, and the orphan response regulator MxaB (Morris & Lidstrom, 1992; Xu et al., 1995; Springer, Morris & Lidstrom, 1997; Springer, Auman & Lidstrom, 1998). The presence of *xoxF1* and *xoxF2* genes are also required for MxaF protein expression in *M. extorquens* AM1 (Skovran et al., 2011). The differential regulation of the *mxaFI* and *xoxF* genes is at least partly carried out by the MxbDM two-component system in *M. extorquens* AM1 (Skovran et al., 2011) and by the orphan response regulator MxaB in *M. buryatense* (Chu & Lidstrom, 2016). These regulators simultaneously increase *mxa* expression while decreasing *xoxF* expression. Like *mxaFI* transcription, transcript levels of *mxaB* are also controlled by lanthanides (Chu & Lidstrom, 2016).

In the non-methanotrophic methylotroph *Paracoccus denitrificans*, the three-gene operon *mxaXYZ* located upstream and divergently transcribed from the *mxaF* operon, was required for growth on methanol (Harms et al., 1993). The response regulator encoded by the *mxaX* gene was shown to be required for *mxaF* gene expression (Yang et al., 1995). Although the histidine kinase encoded by *mxaY* was dispensable for *mxaF* gene expression, the expression of a plasmid-based copy of *mxaY* resulted in enhanced *mxaF* gene transcription (Yang et al., 1995).

*M. buryatense* strain 5GB1C is an attractive model for the study of the lanthanide-mediated MDH switch. It is a fast-growing Type I methanotroph with numerous genetic tools, making it a desirable platform for development as an industrial microorganism (Puri et al., 2015; Yan et al., 2016). Unlike many other methylotrophic organisms that harbor multiple copies of the MDH genes, the *M. buryatense* genome contains only one copy of genes encoding MxaFI and XoxF (Khmelenina et al., 2013). This allows us to easily manipulate and study potential factors involved in the lanthanide-mediated MDH switch.

In this study, we identified an MxaY homologue in *M. buryatense*, which functions to increase the transcription of *mxaB* and the *mxaF* operon while downregulating *xoxF* transcription in the absence of lanthanum. We identified a single nucleotide polymorphism (SNP) in MxaY which renders the histidine kinase MxaY constitutively active. Phenotypic analyses of the *M. buryatense* Δ*mxaY* mutant suggest that the histidine kinase plays pleiotropic roles in lanthanide-mediated cellular response.

## Materials and Methods

### Strains and growth conditions

*M. buryatense* 5GB1C and its derivatives were grown in a modified NMS medium with methane, as previously described (Puri et al., 2015). Supplements were added as follows: 2.5% (weight/volume) sucrose, 100 µg hygromycin/ml, 50 µg kanamycin/ml, 30 µg zeocin/ml, and 30 µM lanthanum chloride (Sigma; lanthanum in 3+ oxidation state). All culturing glassware for experiments performed without lanthanum was acid-washed overnight in 1 M hydrochloric acid before use, which we have shown is sufficient to prevent a lanthanide cellular response (Chu & Lidstrom, 2016). All strains used in this study are listed in Table 1. The *M. buryatense* 5G genome sequence is deposited in GenBank/EMBL under the accession numbers AOTL01000000, KB455575, and KB455576 (Khmelenina et al., 2013).

**Table 1:** *M. buryatense* 5GB1C strains and plasmids used in this study.

Strain name	Genotype	Antibiotic resistance
*M. buryatense* 5GB1C	Wild-type	None
FC53	Δ*xoxF* (Chu & Lidstrom, 2016)	Unmarked
FC57	Δ*xoxF*S::zeo^R^ (Chu & Lidstrom, 2016)	Zeocin
FC70	ΔMETBUDRAFT_1817::zeo^R^	Zeocin
FC74	Δ*mxaY*	Unmarked
FC77	Δ*mxaY*; METBUDRAFT_2794::P_*mxaY*_-*mxaY*	Unmarked
FC78	Δ*mxaY*; METBUDRAFT_2794::P_*mxaY*_-*mxaY* E147G	Unmarked
FC93	Δ*xoxF*S Δ*mxaY* E147G; METBUDRAFT_2794::P_*mxaY*_-*mxaY*	Unmarked
Plasmids		
pFC44	METBUDRAFT_2794::P_*mxaY*_-*mxaY*	Kanamycin
pFC45	METBUDRAFT_2794::P_*mxaF*_-*mxaY* E147G	Kanamycin

### DNA isolation and whole genome resequencing of Δ*xoxF*S

The Δ*xoxF*S strain was isolated as previously described (Chu & Lidstrom, 2016). Genomic DNA from this strain was isolated using a phenol chloroform extraction. The genome resequencing was performed on an Illumina MiSeq with Illumina’s 300-bp paired-end (PE) protocol by MR DNA lab (Shallowater, TX, USA) with multiplexing. The paired-end reads for each condition were pooled and processed with breseq version 0.27.1 (Deatherage & Barrick, 2014) against the MaGE (Vallenet et al., 2013) MBURv2 annotation of *M. buryatense* 5G from 26 November 2013.

### Genetic manipulations

Gene knockout constructs were composed of assembled PCR products and electroporated into *M. buryatense*5GB1C as previously described (Chu & Lidstrom, 2016; Yan et al., 2016). For construction of the final *ΔmxaY* mutant and Δ*xoxFS* Δ*mxaY* mutant, the zeocin resistance marker was excised, leaving an unmarked *mxaY* deletion with a single flippase (FLP) recombinase target (FRT) site remaining, using the FLP-FRT site-specific recombination system (Chu & Lidstrom, 2016).

All complementation strains were constructed by conjugation of a pCM433kanT-based suicide plasmid harboring P_*mxaY*_ and a wild-type or E147G mutant copy of the *mxaY* open reading frame, into the appropriate knockout strain (Puri et al., 2015). Conjugation was performed using *Escherichia coli* S17-1 *λ*pir as the donor strain (Puri et al., 2015). This complementation method results in the integration of the *mxaY* construct into a region of the *M. buryatense* 5GB1C chromosome known to be transcriptionally silent (between genes METBUDRAFT_2794 and METBUDRAFT_2795), allowing for native transcription of the inserted *mxaY* gene (Yan et al., 2016). Sucrose counter-selection was employed to unmark all complementation constructs (Puri et al., 2015). Strain FC74 was conjugated with plasmid pFC44 to create the wild-type *mxaY* complementation strain (FC77) or with plasmid pFC45 to create the E147G MxaY complementation strain (FC78). Strain FC57 was conjugated with plasmid pFC44 to insert a wild-type copy of *mxaY* into the Δ*xoxF*S strain prior to deleting the native copy of *mxaY*, and creating the final strain FC93 (Δ*xoxF*S Δ*mxaY*; METBUDRAFT_2794::P_*mxaY*_-*mxaY*).

Plasmids were constructed by Gibson assembly (Gibson et al., 2009). The plasmid pFC45 containing the *mxaY* E147G construct was constructed using Gibson assembly with a PCR product containing the *mxaY* E147G gene. The *mxaY* E147G PCR product was made by fusion of PCR products with primers containing the E147G mutation (A440G nucleotide mutation) (Shevchuk et al., 2004). All strains and plasmids are listed in Table 1. All primers are listed in Table S1.

### RNA isolation and real-time qRT-PCR assays

RNA was isolated and checked for purity as previously described (Chu & Lidstrom, 2016). Briefly, cells were grown in the presence or absence of lanthanum and RNA was harvested using an acid phenol-chloroform extraction. We used iScript™ Reverse Transcription Supermix (Biorad) with and without reverse transcriptase to ensure all RNA was DNA-free. RNA quality and concentration was determined using Nanodrop^®^ ND-1000. cDNA was generated using 100 ng–500 ng isolated RNA as template with the SensiFAST™ cDNA Synthesis Kit (Bioline). PCR reactions contained the following components: 400 µM primers, SensiFAST™ SYBR^®^ No-ROX Kit (Bioline), cDNA, and ddH_2_O up to 10 µl volume. The PCR reactions were performed in LightCycler^®^ Capillaries (Roche Diagnostics) and reactions were run using a LightCycler 2.0 (Roche Diagnostics). The following PCR program was used: Step 1: 95°C for 3 min, Step 2: 95°C for 10 s, Step 3: 55°C for 15 s, Step 4: 72°C for 45 s, Step 5: 72°C for 3 s (SYBR detection), Step 6: repeat steps 2 through 5 40 times. Ct values were determined using LightCycler Software Version 3.5 (Roche) and all gene expression values were normalized to 16S Ct values. All primers used for real-time quantitative reverse transcription (qRT)-PCR assays are listed in Table S1.

## Results

### Δ*xoxF*S mutant contains mutation in gene encoding MxaY

**Figure 1:**
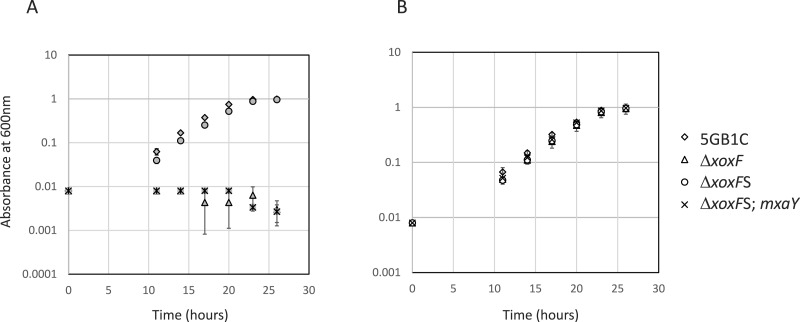
Mutation in MxaY is responsible for Δ*xoxF*S suppressor phenotype. Growth curves for wild-type *M. buryatense* 5GB1C and Δ*xoxF*(FC53), Δ*xoxF*S (Δ*xoxF* suppressor strain; FC57), and Δ*xoxF*S complemented with a wild-type copy of *mxaY* (Δ*xoxF*S; *mxaY*; FC93) grown in cultivation medium with (A, filled symbols) and without (B, empty symbols) 30 µM lanthanum. Data points represent the mean from three replicates and error bars represent standard deviation.

Previously, we identified a suppressor mutant of the *M. buryatense* 5GB1C Δ*xoxF* strain, named Δ*xoxF*S (Chu & Lidstrom, 2016). This suppressor mutant displayed a wild-type growth rate when cultivated in the presence of lanthanum, conditions under which the MxaFI MDH is normally not produced (Fig. 1). We identified the Δ*xoxF*S suppressor mutant allowed for the constitutive activation of the operon encoding MxaFI as well as its transcriptional regulator, MxaB. We submitted the Δ*xoxFS* mutant for whole genome sequencing (WGS) to attempt to identify the causative mutation of the broken lanthanide switch. In the Δ*xoxFS* mutant, we identified a mutation in the gene encoding a histidine kinase (locus tag METBUDRAFT_1818) that would convert a glutamate at amino acid positon 147 to a glycine. The mutated histidine kinase contains homology to genes encoding the MxaY histidine kinase. The histidine kinase in *M. buryatense* shares a 27% amino acid identity to *P. denitrificans* MxaY, 40% identity to *Methylococcus capsulatus* MxaY, and 51% identity to *Methyloglobulus morosus* annotated MxaY. The glutamic acid at amino acid position 147 is widely conserved, but lies outside of the domains involved in histidine kinase function (Galperin & Nikolskaya, 2007; Camacho et al., 2009). Interestingly in the *P. denitrificans* genome, the location of the *mxaXYZ* operon, upstream and divergently transcribed from the *mxaF* operon, is the same as the location of the *mxaB* gene in the *M. buryatense* genome.

To determine if the E147G mutation in MxaY is the causative mutation in the Δ*xoxFS* strain, a wild-type copy of *mxaY* under the control of its native promoter was inserted into the Δ*xoxFS* chromosome at a location known to be transcriptionally silent (Yan et al., 2016). The existing copy of *mxaY* (containing the E147G mutation) was then deleted in this strain. The resulting strain (FC93) contains the Δ*xoxF* and Δ*mxaY* E147G deletions from their native locations and a heterologously expressed copy of wild-type *mxaY*. We determined the growth rate of the resulting *mxaY* complemented strain, compared to Δ*xoxFS* and the original Δ*xoxF* strain. The restoration of one wild-type copy of *mxaY* to the original Δ*xoxF*S strain resulted in a severe growth rate defect when the strain was grown with lanthanum (Table 2 and Fig. 1). This phenotype resembled that of the original Δ*xoxF* strain (Table 2 and Fig. 1). In contrast, the Δ*xoxF*S strain demonstrated a wild-type growth rate regardless of the presence or absence of lanthanum (Table 2 and Fig. 1) (Chu & Lidstrom, 2016). These results demonstrate that the MxaY E147G mutation was responsible for the suppressor phenotype observed in the Δ*xoxF*S strain.

**Table 2:** Doubling times of *xoxF* variant strains.^a^

Strain	With La^3+^	Without La^3+^
WT 5GB1C	2.32 ± 0.17	2.75 ± 0.17
Δ*xoxF*	0 ± 0	2.63 ± 0.15
Δ*xoxF*S	2.24 ± 0.19	2.52 ± 0.14
Δ*xoxF*S Δ*mxaY* E147G; P_*mxaY*_-*mxaY*	0 ± 0	2.58 ± 0.33

**Notes.**

a Doubling times are in hours and represent the means of three technical replicates ± standard deviation. Doubling times were calculated from three time points during the exponential phase of growth, when possible.

TITLE WT wild-type La^3+^ lanthanum

### MxaY controls the lanthanide-mediated MDH switch

**Figure 2:**
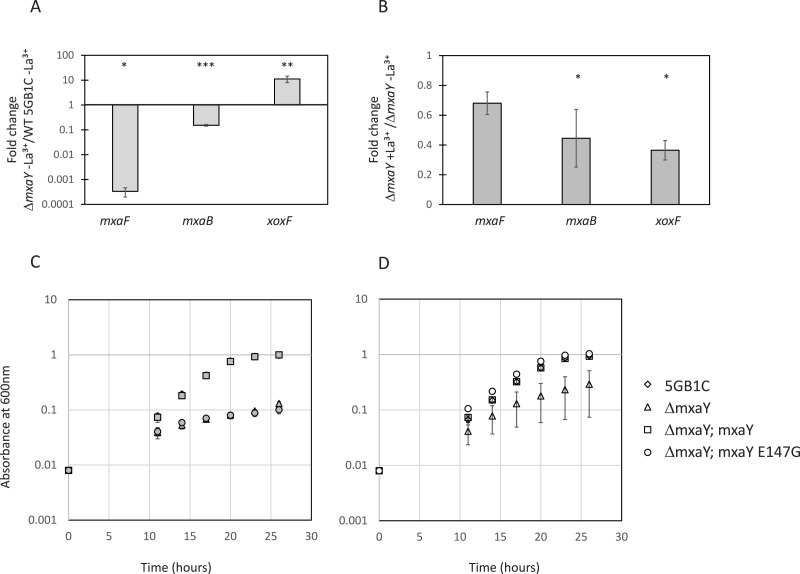
MxaY regulates the lanthanide-mediated MDH switch. (A) Real-time qRT-PCR was performed on RNA harvested from wild-type and Δ*mxaY*(FC74) *M. buryatense* 5GB1C strains grown in the absence of lanthanum. The values shown represent the fold change in *mxaF*, *mxaB*, and *xoxF* gene expression when transcript levels from the Δ*mxaY* strain were compared to wild-type *M. buryatense* 5GB1C. (B) Real-time qRT-PCR was performed on RNA harvested from the Δ*mxaY* mutant grown with and without lanthanum. Results shown represent the fold change in gene expression in Δ*mxaY* cells grown with lanthanum compared to gene expression in Δ*mxaY* cells grown without lanthanum. All gene expression was normalized to 16S rRNA transcript levels. Significant differences between gene expression levels in (A) and (B) were determined by using unpaired *t*-tests (^∗∗∗^*p* < 0.001; ^∗∗^*p* < 0.01; ^∗^*p* < 0.05). La^3+^, lanthanum. (C, D) Growth curves for wild-type *M. buryatense* 5GB1C, Δ*mxaY* (FC74), Δ*mxaY* complemented with a wild-type version of *mxaY* (Δ*mxaY*; mxaY; FC77), and Δ*mxaY* complemented with the E147G version of *mxaY* (Δ*mxaY*; mxaY E147G; FC78) strain variants grown in the presence (C, filled symbols) or absence (D, empty symbols) of 30 µM lanthanum. Data represent the means from three replicates and error bars represent standard deviations.

The *mxaY* gene encodes a histidine kinase whose homolog has been previously shown to be involved in *mxaF* regulation in *P. denitrificans* (Harms et al., 1993; Yang et al., 1995). In order to determine whether MxaY played a role in the lanthanide-mediated MDH switch in *M. buryatense* 5GB1C, an *mxaY* deletion mutation was generated. Real-time qRT-PCR was performed on RNA isolated from the Δ*mxaY* strain. Compared to wild-type *M. buryatense*5GB1C grown in the absence of lanthanum (when *mxa* operon transcription is normally active), the Δ*mxaY* strain grown in the absence of lanthanum shows a decrease in *mxaF* gene transcription and an increase in *xoxF* transcripts (Fig. 2A). The Δ*mxaY* phenotype is similar to that of the Δ*mxaB* mutant (Chu & Lidstrom, 2016), and indicates that MxaY also functions to upregulate transcripts for the MxaFI-type MDH and repress transcripts for the XoxF-type MDH. We also observed a decrease in the level of transcripts from the lanthanide-responsive *mxaB* in the Δ*mxaY* mutant compared to wild-type *M. buryatense* 5GB1C, indicating that MxaY also induces transcription of the orphan response regulator. The results from the Δ*mxaY* mutant strain suggest that MxaY is normally active when cells are cultivated in the absence of lanthanum.

When transcripts from Δ*mxaY* mutant cells grown with lanthanum are compared to transcripts from Δ*mxaY* cells grown in the absence of lanthanum, the lanthanide-mediated *mxa* and *xoxF* transcriptional regulation disappears (Fig. 2B). Interestingly, *xoxF* transcription is not upregulated in a lanthanide-dependent manner in the Δ*mxaY* mutant. This is in contrast to the Δ*mxaB* mutant, in which we still observed a 3X increase in *xoxF* expression in the presence compared to the absence of lanthanum (Chu & Lidstrom, 2016). These results altogether suggest that MxaY functions upstream of MxaB in the regulatory pathway that controls the lanthanide-mediated MDH switch in *M. buryatense*. At this time, it is unclear whether MxaY phosphorylates MxaB directly or serves as part of a more complex regulatory cascade.

The Δ*mxaY* strain displayed impaired growth in the presence or absence of lanthanum (Table 3, Figs. 2C and 2D). When cultured in the absence of lanthanum, the Δ*mxaY* mutant has downregulated *mxa* transcripts and there is no lanthanum to activate XoxF MDH function. Interestingly, some Δ*mxaY* cultures still grew to high cell densities in the absence of lanthanum, although there was great disparity in the growth rate of the replicates (Table 3 and Fig. 2D). In the presence of lanthanum the Δ*mxaY* mutant still exhibits a severe growth defect (Table 3 and Fig. 2C), which suggests that MxaY has a pleiotropic effect on lanthanide-controlled metabolism. The Δ*mxaY* phenotype can be complemented by the addition of a chromosomally expressed copy of the wild-type *mxaY* gene under the control of its native promoter (Fig. S1, Table 3).

**Table 3:** Doubling times of *mxaY* variant strains.^a^

Strain	With La^3+^	Without La^3+^
WT 5GB1C	2.44 ± 0.11	2.66 ± 0.35
Δ*mxaY*	7.63 ± 1.87	4.78 ± 2.46
Δ*mxaY*; P_*mxaY*_-*mxaY*	2.38 ± 0.16	2.80 ± 0.01
Δ*mxaY*; P_*mxaY*_-*mxaY* E147G	7.47 ± 1.37	2.91 ± 0.07

**Notes.**

a Doubling times are in hours and represent the means of three technical replicates ± standard deviation. Doubling times were calculated from three time points during the exponential phase of growth, when possible.

TITLE WT wild-type La^3+^ lanthanum

Located downstream of *mxaY* is the gene, METBUDRAFT_1817, which is predicted to encode a response regulator. The protein product of gene METBUDRAFT_1817 is 50% identical to *M. buryatense* MxaB at the amino acid level. However, METBUDRAFT_1817 does not have significant homology to the response regulator MxaX, which in *P. denitrificans* is encoded by the gene downstream of *mxaY* and is required for *mxaF* gene transcription in Harms et al. (1993); Yang et al. (1995). Because of its location in the *M. buryatense* genome and its similarity to MxaB, we tested whether METBUDRAFT_1817 was involved in the lanthanide-mediated MDH switch. A deletion mutation in this gene did not alter the lanthanide-mediated regulation of *mxa* genes or *xoxF* compared to wild-type *M. buryatense* 5GB1C (Fig. S2) (Chu & Lidstrom, 2016).

### MxaY E147G mutant is constitutively active

**Figure 3:**
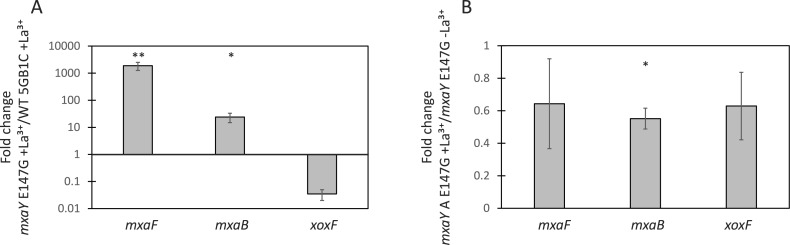
MxaY E147G is a constitutively active version of MxaY. (A) Real-time qRT-PCR was performed on RNA harvested from wild-type and MxaY E147G (FC78) *M. buryatense* 5GB1C strains grown in the presence of 30 µM lanthanum. The values shown represent the fold change in gene expression in MxaY E147G compared to wild-type *M. buryatense* 5GB1C. (B) Real-time qRT-PCR was performed on RNA harvested from MxaY E147G (FC78) cells grown in the presence or absence 30 µM lanthanum. Results represent the fold change in gene expression in cells grown with lanthanum compared to cells grown without lanthanum. All gene expression was normalized to 16S rRNA transcript levels. Significant differences between gene expression levels was determined by using unpaired *t*-tests (^∗∗^*p* < 0.01; ^∗^*p* < 0.05). Data represent the means from three replicates and error bars represent standard deviations. La^3+^, lanthanum.

To determine the consequence of the causative *mxaY* suppressor mutation identified in the Δ*xoxFS* strain, a strain was constructed that contained only the E147G mutant version of MxaY. This strain was generated from the Δ*mxaY* mutant by integrating the gene encoding MxaY E147G at a heterologous site in the chromosome, under the control of the *mxaY* promoter. Real-time qRT-PCR was performed on RNA isolated from the MxaY E147G strain grown in the presence or absence of lanthanum. Compared to wild-type *M. buryatense* 5GB1C grown in the presence of lanthanum, conditions under which we predict MxaY is normally inactive, the MxaY E147G strain grown with lanthanum displays increased expression of *mxaF* and *mxaB* and decreased *xoxF* expression (Fig. 3A). The MxaY E147G strain exhibited similar levels of *mxaF*, *mxaB*, and *xoxF* expression regardless of the presence or absence of lanthanum (Fig. 3B). Together, these results suggest that the E147G mutation renders MxaY constitutively active.

In agreement with the above results, the MxaY E147G mutant has a wild-type growth rate when cultivated in the absence of lanthanum (Fig. 2D and Table 3), conditions under which we predict *mxaY* is normally activated. The MxaY E147G mutant surprisingly displayed a marked growth defect when grown in the presence of lanthanum (Fig. 2C and Table 3). This indicates that inappropriate inactivation of MxaY is detrimental to the cell.

## Discussion

The transcription of genes encoding the MxaFI and XoxF MDH systems is tightly and differentially controlled by a lanthanide-mediated switch (Farhan Ul Haque et al., 2015b; Chu & Lidstrom, 2016; Gu et al., 2016; Vu et al., 2016). In this paper, we describe another regulator of the lanthanide-mediated switch, MxaY, that transcriptionally upregulates genes encoding the MxaFI MDH while downregulating *xoxF* in *M. buryatense*. MxaY is the most upstream factor in the lanthanide-regulatory cascade identified to date, as it also controls the transcription of the MxaB response regulator (Figs. 2A and 2B). It is not yet known whether MxaY and MxaB interact directly, or whether they are part of a more complex signaling cascade. Our findings are in contrast to the MDH regulatory cascade in *P. denitrificans*, in which a deletion of *mxaY* still allowed for normal *mxaF* transcription, perhaps because of a redundant histidine kinase (Yang et al., 1995). However, MxaY in *P. denitrificans* does play a role in activating *mxaF* expression as overexpression of MxaY resulted in increased *mxaF* transcription (Yang et al., 1995). In *M. buryatense*, MxaY seems to be the predominant histidine kinase regulating the lanthanide-mediated MDH switch, as the lanthanum response of *mxaF* and *xoxF* transcription was absent in the Δ*mxaY* mutant (Fig. 2B).

The growth rate of the Δ*mxaY* mutant is impaired when cultivated in both the presence and absence of lanthanum (Figs. 2C and 2D; Table 3). Previous studies have shown that MDH mutants grow at wild-type growth rates under cultivation conditions in which the alternative MDH is transcribed and active (Farhan Ul Haque et al., 2015a; Chu & Lidstrom, 2016; Vu et al., 2016). These growth rate results suggest MxaY function affects more than just MDH gene expression. In addition, the extent of the growth rate impairment is lanthanide-mediated, indicating that MxaY plays a larger role in the lanthanum response than just *mxaFI* and *xoxF* transcription. The identification of the MxaY regulon may aid in the discovery of other lanthanide-responsive genes, which may or may not be involved in MDH function and regulation.

The growth rate of Δ*mxaY* cultivated in the absence of lanthanum was higher than expected (Fig. 2D and Table 3), as *mxaFI* genes are downregulated and no supplemented lanthanum is present for the XoxF active site under these conditions. Several possible explanations exist for this observation. First, *M. buryatense* 5GB1C may be able to scavenge minute amounts of lanthanides present in the media or glassware to support limited growth. Second, alternative metals, such as calcium may be able to poorly substitute for lanthanides in the XoxF active site. Last, the high standard deviation of the Δ*mxaY* mutant growth rate in the absence of lanthanum could point to the presence of spontaneous suppressors arising. It is possible these suppressor mutations may allow XoxF to better utilize alternative metals in its active site.

Our results suggest the identified MxaY E147G mutation allows for the constitutive activation of MxaY, regardless of the presence or absence of lanthanum (Fig. 3). As this amino acid residue lies outside of the domains required for histidine kinase function but is highly conserved amongst methylotrophic homologues, it is tempting to speculate this E147 residue is involved in the lanthanide response. The suppressor mutants of the *M. buryatense* 5GB1C Δ*xoxF* mutant arose easily (Chu & Lidstrom, 2016), and perhaps this means multiple mutations in MxaY can result in constitutive activation. If MxaY is directly sensing lanthanum, E147 and other potential residues identified from Δ*xoxF*S variants may lead to the identification of a lanthanide-binding motif.

The constitutive activation of MxaY in the MxaY E147G strain resulted in a growth rate defect when the strain was cultivated in the presence of lanthanum (Fig. 2C and Table 3). Curiously, the Δ*xoxF*S strain also contains a constitutively activated MxaY copy, but has a wild-type growth rate in the presence of lanthanum (Fig. 1A and Table 2) (Chu & Lidstrom, 2016). In strains with MxaY constitutively activated, genes encoding MxaFI will be transcribed and the enzyme active because of the presence of calcium in the cultivation medium. The growth rate results suggest the additional presence of an active XoxF MDH, which is present in the MxaY E147G complementation strain but not in Δ*xoxF*S, is detrimental to the cell. Despite the fact that MxaY represses XoxF, some active XoxF is predicted to be present in the MxaY E147G strain, since we previously have shown that in an Δ*mxaF* mutant the repressed level of XoxF in the absence of supplemental lanthanum allows for some growth (Chu & Lidstrom, 2016). Our results suggest that this level of active XoxF, combined with active MxaFI result in a drastic reduction in growth rate (Fig. 2C and Table 3). This is the first evidence suggesting that tight control of the MDH enzymes is required for proper metabolic function. Uncontrolled activation of both MDH enzymes might be detrimental to growth if the high levels of transcription and translation of the MDH components places too great a burden on cells.

## Conclusion

The use of methanotrophs as industrial microorganisms is an attractive method of producing value-added chemicals while utilizing excess atmospheric methane. The economic feasibility of this approach depends upon successful metabolic engineering strategies, which in turn depends on an in-depth knowledge of methanotrophic metabolism and its regulation. The expression of the two forms of MDH is an example of regulation that could have an impact on metabolic engineering. Here we identified an additional regulator, the histidine kinase MxaY, of the lanthanide-mediated MDH switch in *M. buryatense*. We identified a SNP located outside of the conserved histidine kinase region that allows for constitutive activation of MxaY. This information could potentially lead to identification of a lanthanide response or binding domain. Currently, there is great interest in studying the role of lanthanides in methylotrophic metabolism and identification of a lanthanide sensor and binding domain may result in the discovery of additional lanthanide-mediated functions in methylotrophic microorganisms.

##  Supplemental Information

10.7717/peerj.2435/supp-1 Figure S1 Complementation of Δ*mxaY* restores lanthanide-mediated MDH gene expression Real-time qRT-PCR was performed on RNA harvested from the Δ*mxaY* wild-type complementation strain (FC77) grown in the presence or absence of 30 µM lanthanum. Results shown represent the fold change in gene expression in cells grown in the presence of lanthanum compared to gene expression in cells grown in the absence of lanthanum. Gene expression was normalized to 16S rRNA transcript levels. Unpaired *t*-tests were used to determine significance in gene expression levels between the two conditions tests (∗∗∗*p* < 0.001, ∗∗*p* < 0.01). Data represent the means from three replicates and error bars represent standard deviations. La^3+^, lanthanum.

10.7717/peerj.2435/supp-2 Figure S2 Predicted response regulator METBUDRAFT_1817 is not required for lanthanide-mediated MDH switch Real-time qRT-PCR was performed on RNA harvested from Δ MBURv2_1817 (FC70) *M. buryatense* 5GB1C cells cultivated in the presence or absence of 30 µM lanthanum. Results shown represent the fold change in gene expression in cells grown in the presence of lanthanum compared to gene expression in cells grown in the absence of lanthanum. Gene expression was normalized to 16S rRNA transcript levels. Unpaired *t*-tests were used to determine significance in gene expression levels between the two conditions tests (∗∗*p* < 0.01). Data represent the means from three replicates and error bars represent standard deviations. La^3+^, lanthanum.

10.7717/peerj.2435/supp-3 Table S1 Primers used in this study

10.7717/peerj.2435/supp-4 Data S1 MxaY raw data Raw data for both growth curves and real-time qRT-PCR assays

## Additional Information and Declarations

### DNA Deposition

The following information was supplied regarding the deposition of DNA sequences: NCBI BioProject Accession number: PRJNA322692.

### Data Availability

The following information was supplied regarding data availability: The raw data has been supplied as a Supplemental Dataset.

## References

[ref-1] Anthony C (1982). The biochemistry of methylotrophs.

[ref-2] Camacho C, Coulouris G, Avagyan V, Ma N, Papadopoulos J, Bealer K, Madden TL (2009). BLAST+: architecture and applications. BMC Bioinformatics.

[ref-3] Chistoserdova L (2011). Modularity of methylotrophy, revisited. Environmental Microbiology.

[ref-4] Chistoserdova L, Lidstrom ME (1997). Molecular and mutational analysis of a DNA region separating two methylotrophy gene clusters in *Methylobacterium extorquens* AM1. Microbiology.

[ref-5] Choi DW, Kunz RC, Boyd ES, Semrau JD, Antholine WE, Han JI, Zahn JA, Boyd JM, De la Mora AM, Dispirito AA (2003). The membrane-associated methane monooxygenase (pMMO) and pMMO-NADH:quinone oxidoreductase complex from *Methylococcus capsulatus* bath. Journal of Bacteriology.

[ref-6] Chu F, Lidstrom ME (2016). XoxF acts as the predominant methanol dehydrogenase in the type I methanotroph *Methylomicrobium buryatense*. Journal of Bacteriology.

[ref-7] Deatherage DE, Barrick JE (2014). Identification of mutations in laboratory-evolved microbes from next-generation sequencing data using breseq. Methods in Molecular Biology.

[ref-8] Delmotte N, Knief C, Chaffron S, Innerebner G, Roschitzki B, Schlapbach R, Von Mering C, Vorholt JA (2009). Community proteogenomics reveals insights into the physiology of phyllosphere bacteria. Proceedings of the National Academy of Sciences of the United States of America.

[ref-9] Farhan Ul Haque M, Gu W, DiSpirito AA, Semrau JD (2015a). Marker exchange mutagenesis of mxaF., encoding the large subunit of the Mxa methanol dehydrogenase, in methylosinus trichosporium OB3b. Applied and Environmental Microbiology.

[ref-10] Farhan Ul Haque M, Kalidass B, Bandow N, Turpin EA, DiSpirito AA, Semrau JD (2015b). Cerium regulates expression of alternative methanol dehydrogenases in methylosinus trichosporium OB3b. Applied and Environmental Microbiology.

[ref-11] Fei Q, Guarnieri MT, Tao L, Laurens LM, Dowe N, Pienkos PT (2014). Bioconversion of natural gas to liquid fuel: opportunities and challenges. Biotechnology Advances.

[ref-12] Fitriyanto NA, Fushimi M, Matsunaga M, Pertiwiningrum A, Iwama T, Kawai K (2011). Molecular structure and gene analysis of Ce3+ -induced methanol dehydrogenase of Bradyrhizobium sp. MAFF211645. Journal of Bioscience and Bioengineering.

[ref-13] Galperin MY, Nikolskaya AN (2007). Identification of sensory and signal-transducing domains in two-component signaling systems. Methods in Enzymology.

[ref-14] Gibson DG, Young L, Chuang RY, Venter JC, Hutchison CA, Smith HO (2009). Enzymatic assembly of DNA molecules up to several hundred kilobases. Nature Methods.

[ref-15] Grob C, Taubert M, Howat AM, Burns OJ, Dixon JL, Richnow HH, Jehmlich N, Von Bergen M, Chen Y, Murrell JC (2015). Combining metagenomics with metaproteomics and stable isotope probing reveals metabolic pathways used by a naturally occurring marine methylotroph. Environmental Microbiology.

[ref-16] Gu W, Haque MF, Dispirito AA, Semrau JD (2016). Uptake and effect of rare earth elements on gene expression in methylosinus trichosporium OB3b. FEMS Microbiology Letters.

[ref-17] Harms N, Ras J, Koning S, Reijnders WNM, Stouthamer AH, Van Spanning RJM, Lidstrom ME, Tabita FR (1996). Genetics of C1 metabolism regulation in *Paracoccus denitrificans*. Microbial Growth on C1 compounds.

[ref-18] Harms N, Reijnders WN, Anazawa H, Van der Palen CJ, Van Spanning RJ, Oltmann LF, Stouthamer AH (1993). Identification of a two-component regulatory system controlling methanol dehydrogenase synthesis in *Paracoccus denitrificans*. Molecular Microbiology.

[ref-19] Haynes CA, Gonzalez R (2014). Rethinking biological activation of methane and conversion to liquid fuels. Nature Chemical Biology.

[ref-20] Hibi Y, Asai K, Arafuka H, Hamajima M, Iwama T, Kawai K (2011). Molecular structure of La3+-induced methanol dehydrogenase-like protein in Methylobacterium radiotolerans. Journal of Bioscience and Bioengineering.

[ref-22] Kalyuzhnaya MG, Puri AW, Lidstrom ME (2015). Metabolic engineering in methanotrophic bacteria. Metabolic Engineering.

[ref-21] Kalyuzhnaya MG, Yang S, Rozova ON, Smalley NE, Clubb J, Lamb A, Gowda GA, Raftery D, Fu Y, Bringel F, Vuilleumier S, Beck DA, Trotsenko YA, Khmelenina VN, Lidstrom ME (2013). Highly efficient methane biocatalysis revealed in a methanotrophic bacterium. Nature Communications.

[ref-23] Keltjens JT, Pol A, Reimann J, Op den Camp HJ (2014). PQQ-dependent methanol dehydrogenases: rare-earth elements make a difference. Applied Microbiology and Biotechnology.

[ref-24] Kemp MB, Quayle JR (1966). Microbial growth on C1 compounds. Incorporation of C1 units into allulose phosphate by extracts of Pseudomonas methanica. Biochemical Journal.

[ref-25] Kemp MB, Quayle JR (1967). Microbial growth on C1 compounds. Uptake of [14C]formaldehyde and [14C]formate by methane-grown Pseudomonas methanica and determination of the hexose labelling pattern after brief incubation with [14C]methanol. Biochemical Journal.

[ref-26] Khmelenina VN, Beck DA, Munk C, Davenport K, Daligault H, Erkkila T, Goodwin L, Gu W, Lo CC, Scholz M, Teshima H, Xu Y, Chain P, Bringel F, Vuilleumier S, Dispirito A, Dunfield P, Jetten MS, Klotz MG, Knief C, Murrell JC, Op den Camp HJ, Sakai Y, Semrau J, Svenning M, Stein LY, Trotsenko YA, Kalyuzhnaya MG (2013). Draft genome sequence of methylomicrobium buryatense strain 5G, a haloalkaline-tolerant methanotrophic bacterium. Genome Announcements.

[ref-27] Lidstrom ME, Balows A, Truper HG, Dworkin M, Harder W, Schleifer KH (2006). Aerobic methylotrophic procaryotes. The prokaryotes.

[ref-28] Morris CJ, Lidstrom ME (1992). Cloning of a methanol-inducible moxF promoter and its analysis in moxB mutants of *Methylobacterium extorquens* AM1rif. Journal of Bacteriology.

[ref-29] Nakagawa T, Mitsui R, Tani A, Sasa K, Tashiro S, Iwama T, Hayakawa T, Kawai K (2012). A catalytic role of XoxF1 as La3+-dependent methanol dehydrogenase in *Methylobacterium extorquens* strain AM1. PLoS ONE.

[ref-30] Nielsen AK, Gerdes K, Murrell JC (1997). Copper-dependent reciprocal transcriptional regulation of methane monooxygenase genes in *Methylococcus capsulatus* and Methylosinus trichosporium. Molecular Microbiology.

[ref-31] Pol A, Barends TR, Dietl A, Khadem AF, Eygensteyn J, Jetten MS, Op den Camp HJ (2014). Rare earth metals are essential for methanotrophic life in volcanic mudpots. Environmental Microbiology.

[ref-32] Puri AW, Owen S, Chu F, Chavkin T, Beck DA, Kalyuzhnaya MG, Lidstrom ME (2015). Genetic tools for the industrially promising methanotroph Methylomicrobium buryatense. Applied and Environmental Microbiology.

[ref-33] Ramachandran A, Walsh DA (2015). Investigation of XoxF methanol dehydrogenases reveals new methylotrophic bacteria in pelagic marine and freshwater ecosystems. FEMS Microbiology Ecology.

[ref-34] Schmidt S, Christen P, Kiefer P, Vorholt JA (2010). Functional investigation of methanol dehydrogenase-like protein XoxF in *Methylobacterium extorquens* AM1. Microbiology.

[ref-35] Semrau JD, Dispirito AA, Yoon S (2010). Methanotrophs and copper. FEMS Microbiology Reviews.

[ref-36] Shevchuk NA, Bryksin AV, Nusinovich YA, Cabello FC, Sutherland M, Ladisch S (2004). Construction of long DNA molecules using long PCR-based fusion of several fragments simultaneously. Nucleic Acids Research.

[ref-37] Skovran E, Martinez-Gomez NC (2015). Microbiology. Just add lanthanides. Science.

[ref-38] Skovran E, Palmer AD, Rountree AM, Good NM, Lidstrom ME (2011). XoxF is required for expression of methanol dehydrogenase in *Methylobacterium extorquens* AM1. Journal of Bacteriology.

[ref-39] Springer AL, Auman AJ, Lidstrom ME (1998). Sequence and characterization of mxaB, a response regulator involved in regulation of methanol oxidation, and of mxaW, a methanol-regulated gene in *Methylobacterium extorquens* AM1. FEMS Microbiology Letters.

[ref-40] Springer AL, Morris CJ, Lidstrom ME (1997). Molecular analysis of mxbD and mxbM, a putative sensor-regulator pair required for oxidation of methanol in *Methylobacterium extorquens* AM1. Microbiology.

[ref-41] Vallenet D, Belda E, Calteau A, Cruveiller S, Engelen S, Lajus A, Le Fevre F, Longin C, Mornico D, Roche D, Rouy Z, Salvignol G, Scarpelli C, Thil Smith AA, Weiman M, Medigue C (2013). MicroScope–an integrated microbial resource for the curation and comparative analysis of genomic and metabolic data. Nucleic Acids Research.

[ref-42] Vu HN, Subuyuj GA, Vijayakumar S, Good NM, Martinez-Gomez NC, Skovran E (2016). Lanthanide-dependent regulation of methanol oxidation systems in *Methylobacterium extorquens* AM1 and their contribution to methanol growth. Journal of Bacteriology.

[ref-43] Xu HH, Janka JJ, Viebahn M, Hanson RS (1995). Nucleotide sequence of the mxcQ and mxcE genes, required for methanol dehydrogenase synthesis in Methylobacterium organophilum XX: a two-component regulatory system. Microbiology.

[ref-44] Yan X, Chu F, Puri AW, Fu Y, Lidstrom ME (2016). Electroporation-based genetic manipulation in type I methanotrophs. Applied and Environmental Microbiology.

[ref-45] Yang H, Reijnders WNM, Van Spanning RJM, Stouthamer AH, Harms N (1995). Expression of the structural mox genes in *Paracoccus denitrificans* follows wild-type regulation in mutants with a deletion in mxaY, the gene encoding the signal sensor. Microbiology.

