# Supplementary figures and images for: MxaY regulates the lanthanide-mediated methanol dehydrogenase switch in Methylomicrobium buryatense
Source: PeerJ. 2016 Sep 7;4:e2435. doi: 10.7717/peerj.2435 (PMC5018670; doi:10.7717/peerj.2435)

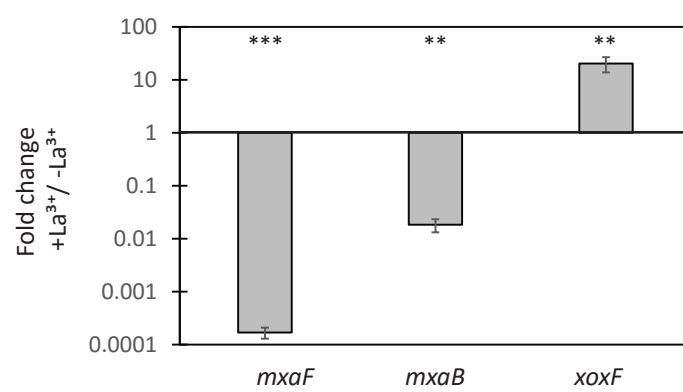

Supplement: Figure S1 — Real-time qRT-PCR was performed on RNA harvested from the ΔmxaY wild-type complementation strain (FC77) grown in the presence or absence of 30 µM lanthanum. Results shown represent the fold change in gene expression in cells grown in the presence of lanthanum compared to gene expression in cells grown in the absence of lanthanum. Gene expression was normalized to 16S rRNA transcript levels. Unpaired t-tests were used to determine significance in gene expression levels between the two conditions tests (∗∗∗p < 0.001, ∗∗p < 0.01). Data represent the means from three replicates and error bars represent standard deviations. La3+, lanthanum. [file peerj-04-2435-s001.pdf]

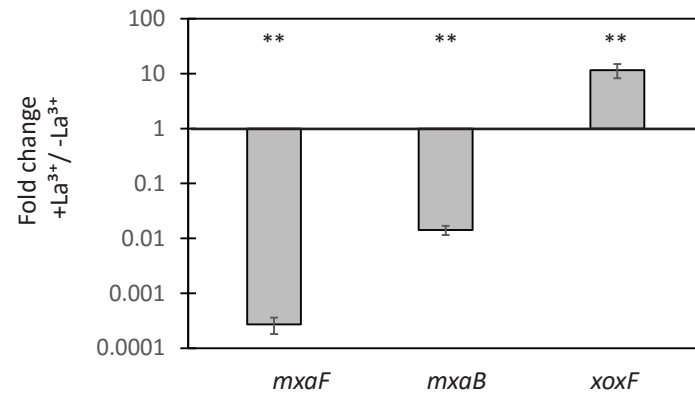

Supplement: Figure S2 — Real-time qRT-PCR was performed on RNA harvested from Δ MBURv2_1817 (FC70) M. buryatense 5GB1C cells cultivated in the presence or absence of 30 µM lanthanum. Results shown represent the fold change in gene expression in cells grown in the presence of lanthanum compared to gene expression in cells grown in the absence of lanthanum. Gene expression was normalized to 16S rRNA transcript levels. Unpaired t-tests were used to determine significance in gene expression levels between the two conditions tests (∗∗p < 0.01). Data represent the means from three replicates and error bars represent standard deviations. La3+, lanthanum. [file peerj-04-2435-s002.pdf]
