# Supplementary material for: MxaY regulates the lanthanide-mediated methanol dehydrogenase switch in Methylomicrobium buryatense
Source: PeerJ. 2016 Sep 7;4:e2435. doi: 10.7717/peerj.2435 (PMC5018670; doi:10.7717/peerj.2435)
Supplement: Table S1 [file peerj-04-2435-s003.docx]

**Table S1**. Primers used in this study*

| For function | Primer name | Sequence |
| --- | --- | --- |
| Gene knockout |  |  |
| FRT-zeo-FRT casette | FC294_zeoR_F | *gaagttcctattctctagaaagtataggaacttc*CTCTGAAATGAGCTGTTGACAATTAATCAT |
|  | FC295_zeoR_R | *gaagttcctatactttctagagaataggaacttc*CGTTCATGTCTCCTTTTTTATTCAGTCCTG |
| Δ*mxaY* | FC541_mxaYLF_F | TTTGTGATGGATTTCCGGTGTGTG |
|  | FC542_mxaYLF_R | tcagag*gaagttcctatactttctagagaataggaacttc*GATCGAACCGCCTAGTAACAATATACA |
|  | FC543_mxaYRF_F | tgaacg*gaagttcctattctctagaaagtataggaacttc*ATGGCAGTTAGCGCAACGATAC |
|  | FC544_mxaYRF_R | CTCGGTGGAAACGAAGAAATTCGA |
| ΔMETBUDRAFT_1817 | FC553_1817LF_F | TTGGCGGTGCATCAAACCTTAC |
|  | FC554_1817LF_R | tcagag*gaagttcctatactttctagagaataggaacttc*GTATCATGCGTTCCGAGCAACA |
|  | FC555_1817RF_F | tgaacg*gaagttcctattctctagaaagtataggaacttc*ACGGCACGCAAATAAGAAATAAGC |
|  | FC556_1817LF_R | GCTCGGCAGTCGTTATACCTTT |
| pFC44 |  |  |
| PmxaY and *mxaY* | FC545_PmxaY_F | ttcatggttaaactgccgaattTTCGACCGAATCGACATATCTCC |
|  | FC546_PmxaYORF_R | tagccatgtttcctcaatggTCATAAAATAGGTATCGTTGCGCTAACT |
| pFC45 |  |  |
| For *mxaY* E147G | FC549_mxaYA440G_F | ATGGATGAAACCGTCGGGGTTTGGCACGAAAC |
|  | FC550_mxaYA440G_R | GTTTCGTGCCAAACCCCGACGGTTTCATCCAT |
| For qRT-PCR |  |  |
| *xoxF* | FC401_276xoxF_F | ATTCACACTCCATTCCCTAACACC |
|  | FC402_550xoxF_R | CTAATGGAGCTTGAGTGTTGGTCATG |
| *mxaF* | FC403_169mxaF_F | AGTTGTACGACATCAACATCACG |
|  | FC404_367mxaF_R | GCTTCGGTTTGAATTGCCACA |
| 16S | FC409_34816S3_F | ATATTGGACAATGGGCGCAAG |
|  | FC410_62316S3_R | CAAATGCCGTTCCCAGGTTAAG |
| *mxaB* | FC413_mxaF_LF_F | AGTTGTTGCGCTAATTCGGGTTC |
|  | FC464_mxaB_R | CAACCGAATATCGTCGTGATGGAC |

*Primer regions used for Gibson or PCR stitching junctions are in lower case, FRT sites are italicized, and *mxaY* site-directed mutation is underlined.
